# Hypoxic metabolism in human hematopoietic stem cells

**DOI:** 10.1186/s13578-015-0020-3

**Published:** 2015-07-17

**Authors:** Fatih Kocabas, Li Xie, Jingjing Xie, Zhuo Yu, Ralph J. DeBerardinis, Wataru Kimura, SuWannee Thet, Ahmed F. Elshamy, Hesham Abouellail, Shalini Muralidhar, Xiaoye Liu, Chiqi Chen, Hesham A. Sadek, Cheng Cheng Zhang, Junke Zheng

**Affiliations:** Department of Internal Medicine, Division of Cardiology, UT Southwestern Medical Center at Dallas, Dallas, TX 75390 USA; Department of Genetics and Bioengineering, Faculty of Engineering, Yeditepe University, Istanbul, 34755 Turkey; Hongqiao International Institute of Medicine, Shanghai Tongren Hospital / Faculty of Basic Medicine, Shanghai Jiao Tong University School of Medicine, Shanghai, 200025 China; Key Laboratory of Cell Differentiation and Apoptosis of Chinese Ministry of Education, Shanghai Jiao Tong University School of Medicine, Chongqing South Road 280, Shanghai, 200025 China; Bingzhou Medical University, Taishan Scholar Program, Yantai, 264003 China; Departments of Pediatrics and Genetics, UT Southwestern Medical Center at Dallas, Dallas, TX 75390 USA; Department of Clinical Pathology, El Galaa Hospital, Cairo, Egypt; Faculty of Medicine Ain Shams University, El Abbaseya, Cairo, Egypt; Departments of Physiology and Developmental Biology, UT Southwestern Medical Center at Dallas, 5323 Harry Hines Blvd, Dallas, TX 75390 USA

**Keywords:** Stem cells, Metabolism, Hypoxia, Hypoxic regulation of metabolism, Human hematopoietic progenitor and stem cells, HPSCs

## Abstract

**Background:**

Adult hematopoietic stem cells (HSCs) are maintained in a microenvironment, known as niche in the endosteal regions of the bone marrow. This stem cell niche with low oxygen tension requires HSCs to adopt a unique metabolic profile. We have recently demonstrated that mouse long-term hematopoietic stem cells (LT-HSCs) utilize glycolysis instead of mitochondrial oxidative phosphorylation as their main energy source. However, the metabolic phenotype of human hematopoietic progenitor and stem cells (HPSCs) remains unknown.

**Results:**

We show that HPSCs have a similar metabolic phenotype, as shown by high rates of glycolysis, and low rates of oxygen consumption. Fractionation of human mobilized peripheral blood cells based on their metabolic footprint shows that cells with a low mitochondrial potential are highly enriched for HPSCs. Remarkably, low MP cells had much better repopulation ability as compared to high MP cells. Moreover, similar to their murine counterparts, we show that Hif-1α is upregulated in human HPSCs, where it is transcriptionally regulated by Meis1. Finally, we show that Meis1 and its cofactors Pbx1 and HoxA9 play an important role in transcriptional activation of Hif-1α in a cooperative manner.

**Conclusions:**

These findings highlight the unique metabolic properties of human HPSCs and the transcriptional network that regulates their metabolic phenotype.

**Electronic supplementary material:**

The online version of this article (doi:10.1186/s13578-015-0020-3) contains supplementary material, which is available to authorized users.

## Background

HSCs are defined by their inherent capacity for self-renewal and differentiation into all blood cell types. Adult HSCs reside in regions of the bone marrow challenged by low oxygen tension (hypoxia), which is termed “hypoxic niche” [1, 2]. We recently demonstrated that mouse HSCs in this hypoxic niche adopt a glycolytic metabolic profile, in which HSCs manifest lower rates of oxygen consumption, lower ATP content, and increased cytoplasmic glycolysis [3]. In addition, we and others showed that mouse HSCs demonstrate increased HIF-1α levels [3–5].

HIF-1 regulates various aspects of metabolism from the oxidant stress response to regulation of glycolysis and mitochondrial respiration [6–12]. HIF-1, a major mediator of transcriptional response to hypoxia, is composed of O_2_ sensitive HIF-1α and constitutively active HIF-1β subunits [13–15]. Conditional deletion of HIF-1α in HSCs results in various defects such as loss of quiescence and progressive decline in HSC number following bone marrow transplantations and aging [4]. In addition hypoxic cultures promote the production of hematopoietic progenitors and enhance HSC expansion [1, 16, 17]. HIF-1α protein is mainly stabilized during hypoxia but normoxic upregulation of HIF-1α has also been reported [14, 18–25]. We recently demonstrated that Meis1 transactivates HIF-1α expression via a conserved Meis1 binding motif located in the first intron of HIF-1α [3], and regulates mouse HSC metabolism [26].

Several reports suggest that homeobox protein Meis1 plays important roles in HPSC biology [27–32]. Homozygous mutant mice for Meis1 die during gestation with defects in hematopoiesis which results in decline in myeloid, lymphoid, and multipotent progenitors [33–35]. Meis1 is also associated with leukemogenesis in humans with a frequent up-regulation in primary acute myeloid leukemia (AML) [29, 30] and acute lymphoblastic leukemia (ALL) samples [36]. We recently showed that mouse LT-HSCs express Meis1 where a great majority of Meis1^+^ HSCs coexpress Hif-1α [3]. Previous reports indicate that Meis1 interacts with the cofactors Pbx1 and HoxA9 in HSCs, which are known regulators of hematopoiesis [27, 28, 37–48]. However, the cooperative role of Meis1 and its cofactors in regulation of HIF-1 is unknown.

In the current report, we demonstrate that human HPSCs have a metabolic profile characterized by low mitochondrial oxidative phosphorylation, dependence on glycolytic metabolism, and upregulation of hypoxia-responsive pathways. We show that the metabolic phenotype of human HPSCs is mediated in part via transcriptional activation of Hif-1α by Meis1 and its cofactors Pbx1 and HoxA9.

## Results

### Metabolic profile of human HPSCs

**Fig. 1:**
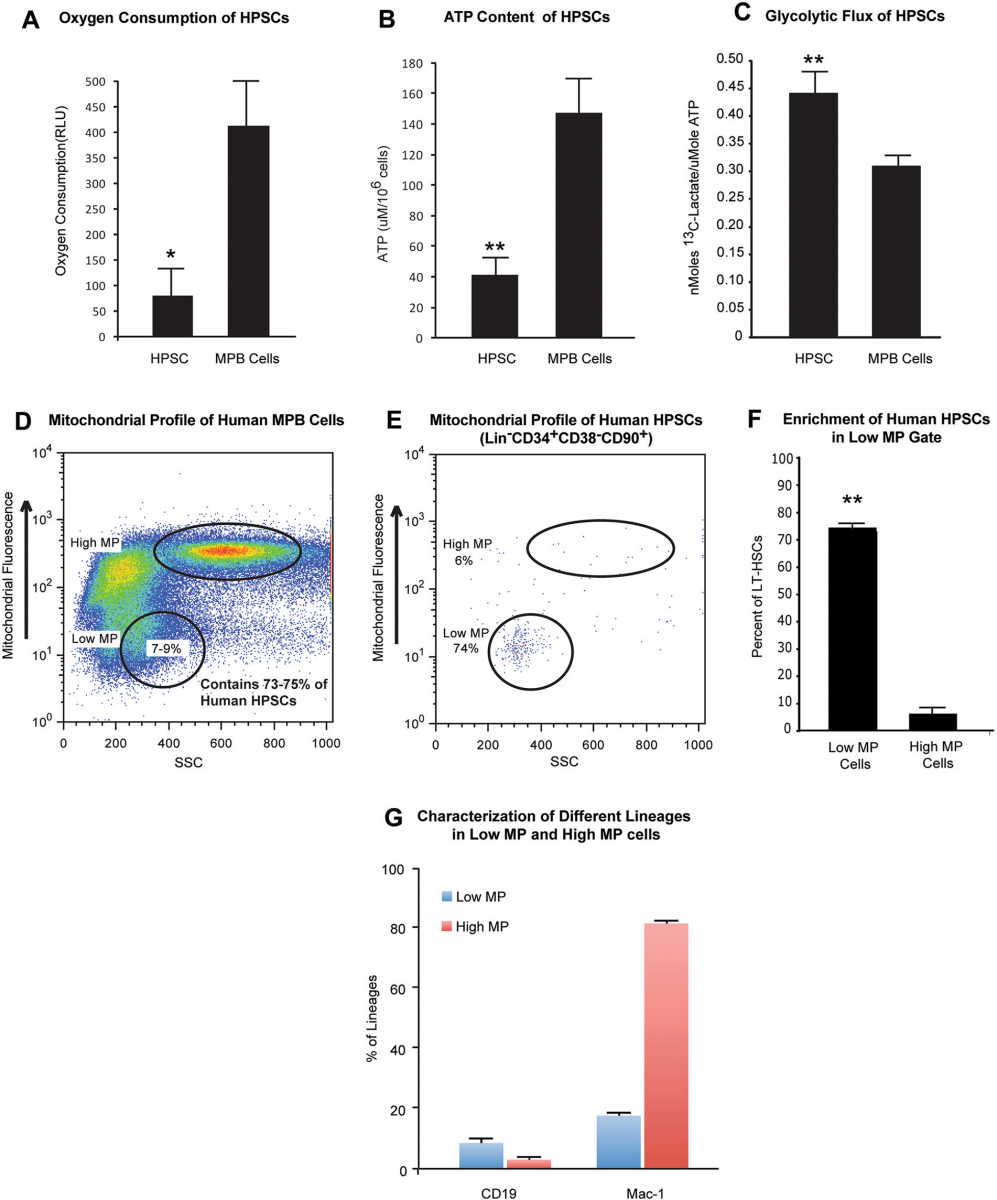
Metabolic profile of human HPSCs. **a**) Oxygen consumption of Lin^−^CD34^+^CD38^−^CD90^+^ HPSCs and human mobilized peripheral blood cells (MPB Cells) demonstrating lower rates of oxygen consumption by HPSCs (*n* = 3). **b**) ATP level of HPSCs and MPB Cells demonstrating lower ATP levels in HPSCs (*n* = 3). **c**) Glycolytic flux of HPSCs and MPB Cells demonstrating higher rates of glycolysis in HPSCs (*n* = 3). **d**) Flow cytometry profile of MPB mononuclear cells stained with mitotracker. Note populations with different mitotracker fluorescence. **e**) Mitotracker profile of human HPSCs. The majority of human HPSCs (73–75 %) are localized to a unique population (7–9 %) of total human MPB mononuclear cells with low mitochondrial potential (low MP). **f**) Quantification of the percent of human HPSCs residing in low MP cells and high MP cells demonstrates significant enrichment of human HPSCs in low MP gate. **g**) Characterization of different lineages within low and high MP cells of MPB. The low MP cells only contained a few B cells (CD19, 7.9 %) and myeloid cells (Mac-1, 17.05 %). The high MP cells had lower percentage of B cells (CD19, 2.4 %), but much higher percentage of myeloid cells (Mac-1, 81.65 %) (*n* =3).*p < 0.05, **p < 0.01

HSCs have been reported to reside in hypoxic niches and this suggested that HSCs require adopting unique metabolic properties. Here, we demonstrate that human HPSCs (Lin^−^CD34^+^CD38^−^CD90^+^ cells) from G-CSF mobilized peripheral blood (MPB) have lower rates of mitochondrial respiration. The overall oxygen consumption by HPSCs is significantly lower compared to MPB mononuclear cells (Fig. 1a). Consistently, human HPSCs have lower ATP content (Fig. 1b). In addition, Fig. 1c shows that the rate of glycolysis is significantly higher in HPSCs compared to the MPB mononuclear cells, suggesting that human HPSCs rely primarily on glycolytic metabolism instead of mitochondrial oxidative phosphorylation.

Next we sought to profile human MPB HPSCs based on their mitochondrial proton gradient as an index of overall mitochondrial respiration. Similar to mouse HSCs, the majority of the human Lin^−^CD34^+^CD38^−^CD90^+^ cells (74 %) fell within a defined flow-cytometry gate which corresponds to 7–9 % of total G-CSF mobilized human peripheral blood cells (Fig. 1d and Additional file 1: Fig. S1a and b). Cells in this gate were characterized by low mitochondrial potential (low MP) while the majority of the remaining MPB populations fell within a high mitochondrial potential (high MP) (Fig. 1e-f). Furthermore, we determined percentages of different lineages within low and high MP cells of MPB (Fig. 1g and Additional file 1: Fig. S1c and d). The results showed the low MP cells only contained a few B cells (CD19, 7.9 %) and myeloid cells (Mac-1, 17.05 %). The high MP cells had lower percentage of B cells (CD19, 2.4 %), but much higher percentage of myeloid cells (Mac-1, 81.65 %). This result indicated that there were less differentiated cells in low MP cells compared to high MP cells, which was consistent with enrichment of HPSCs in low MP cells.

### Low MP cells are primed for hypoxic niche

**Fig. 2:**
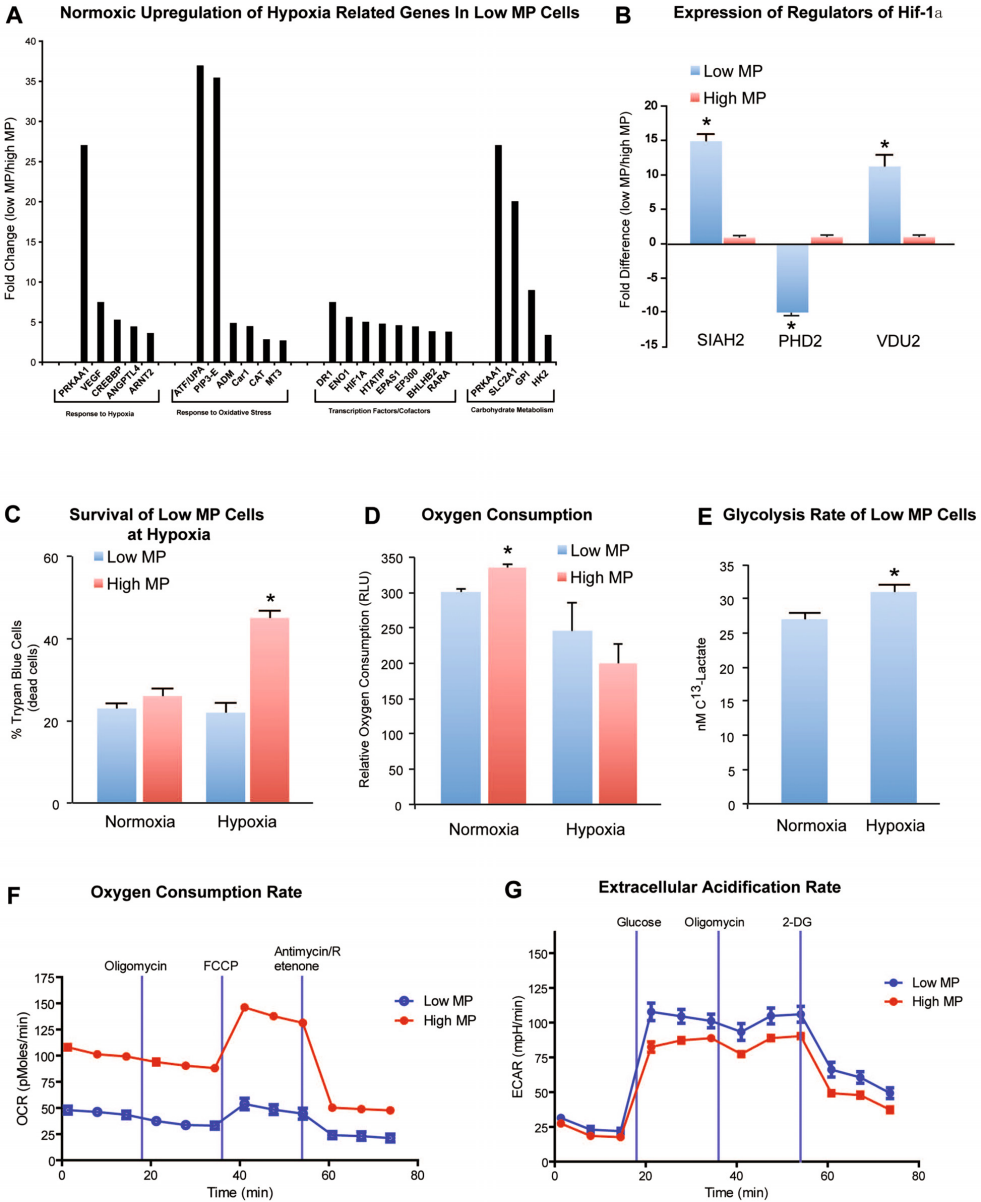
Low MP Cells are primed for hypoxic niche. **a**) Real-time PCR array of low MP cells compared to high MP cells shows significant upregulation of a number of hypoxia inducible genes in the low MP cells at normoxia. **b**) Expression of regulators of Hif-1α in low and high MP cells by RT-PCR. Note the significant downregulation of the Hif-1α destabilizing enzyme PHD2 and the upregulation of two Hif-1α stabilizing enzymes SIAH2 and VDU2 in the low MP cells. **c**) Survival of low MP cells under hypoxia: The viability of low MP cells under hypoxic condition, but not normoxic condition, was much higher than that of high MP cells. **d**) Oxygen consumption by Low MP and High MP cells at normoxia and hypoxia. Low MP cells had lower rates of oxygen consumption at normoxia compared to high MP cells, but no significant difference at hypoxia. **e**) Increased glycolysis rate of low MP cells in vitro measured at hypoxia. Increased glycolytic rate under hypoxia by low MP cells suggest that low MP cells could respond to hypoxia via increasing glycolysis, which indicated the low MP cells prefer glycolysis as the energy source in the physiologic hypoxic niche. **f**) Plots of oxygen consumption rate (OCR) as a parameter of time in low MP cells and high MP cells. **g**) Extracellular acidification rate (ECAR) was plotted as a parameter of time in low MP cells and high MP cells (*n* =3).*p < 0.05

To characterize the metabolic properties of cells in the low and high MP gates, we performed a PCR array for hypoxia related gene expression in freshly isolated low and high MP cells. Under normoxic conditions, the low MP cells were characterized by significant upregulation of numerous hypoxia-inducible genes and regulatory glycolysis genes including Hif-1α (Fig. 2a). These results are not only supportive of the metabolic phenotype of low MP cells, but also suggest that the low MP cells are primed to handle hypoxic stresses. In addition, expression profile of the Hif-1α regulatory genes is in favor of stabilization of Hif-1α protein in low MP cells (Fig. 2b). PHD2, which is responsible for hydroxylation of Hif-1α resulting in its ubiquitination and degradation, was significantly (*p* < 0.05) downregulated in the low MP cells. Conversely, SIAH2 and VDU2, two stabilizers of Hif-1α were significantly (*p* < 0.05) upregulated in low MP cells compared to high MP cells. These results support the higher levels of Hif-1α and the higher expression of Hif-1α target genes in low MP cells.

Increased Hif-1α expression in low MP cells suggests that low MP cells could confer survival advantage at hypoxia. Thus, we measured the viability of low MP and high MP cells at normoxia and hypoxia (Fig. 2c). Survival of low MP cells at hypoxic condition, but not at normoxic condition, was much higher than that of high MP cells. The increased survival under hypoxic conditions by low MP cells in vitro suggested that low MP cells in physiological hypoxia could have higher survival ability. To further confirm this, we examined the apoptotic status of low and high MP cells at different conditions by staining with anti-Annexin V antibodies. Consistently, we demonstrated that there was a dramatic increase of apoptosis in high MP cells at hypoxic condition, but not at normoxic condition (Additional file 2: Fig. S2a). In addition, low MP cells consumed less oxygen compared to high MP cells at normoxia while there is no significant difference at hypoxia (Fig. 2d). Moreover, measurement of glycolysis rate of low MP and high MP cells showed that the glycolytic rate is significantly increased under hypoxia, which indicated the low MP cells preferred to glycolysis as the energy source in the physiologic niche (Fig. 2e). Moreover, we used the Seahorse XF96 extracellular flux analyzer to measure the oxygen consumption rate (OCR) indicative of oxidative phosphorylation, and the lactate production indicative of glycolysis (extracellular acidification rate, ECAR). As shown in Fig. 2f and g, low MP cells showed a significantly lower OCR rate as compared to high MP cells. In contrast, low MP cells showed a significantly higher ECAR rate, indicative of higher glycolytic metabolism. This data further revealed low MP cells using glycolysis as primary energy source.

Together, these results indicate that low MP cells are characterized by baseline upregulation of glycolytic and hypoxia inducible genes, which suggests adaptation of human HPSCs to glycolysis as the major source of energy production.

### Characterization of stem cell profile of metabolically sorted cells

**Fig. 3:**
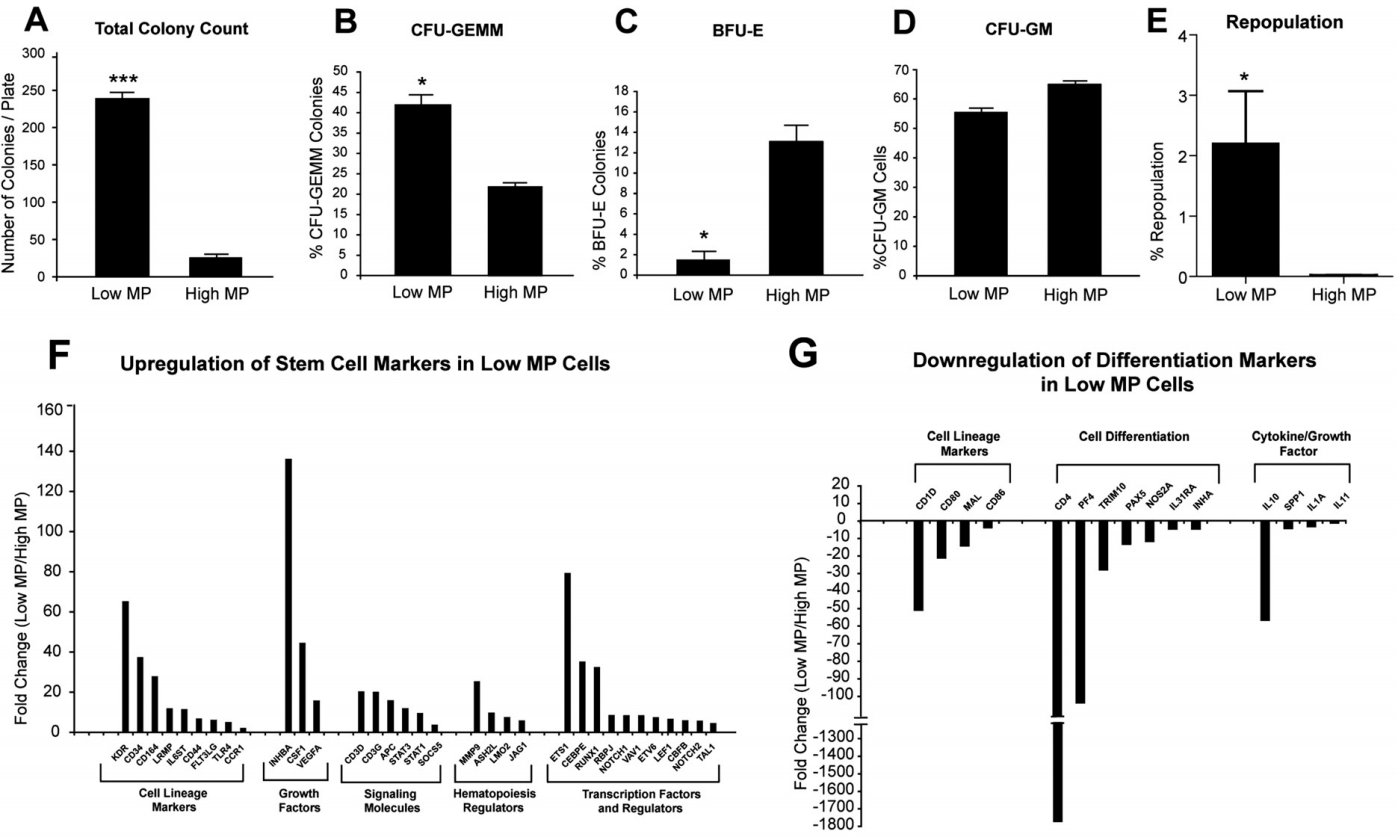
Low MP Cells are enriched for hematopoietic stem cells. In vitro colony forming assay using low and high MP human MPB cells. Panel **a** to **d** shows the number of colonies derived from low and high MP cells after 12 days in methocult medium. Note the significantly higher number of colonies derived from the low MP population. (*n* = 3, *p* < 0.001) Different types of colonies derived from low and high MP cells were analyzed. **b**, **c** and **d** are the percentages of CFU-GEMM, BFU-E and CFU-GM respectively. **e**) FACS-sorted low MP cells and high MP cells were injected into the tibia of sublethally irradiated NOD/SCID mice. The donor repopulation was shown after 2 months. **f**) Real Time PCR array of HPSC markers comparing low MP cells to high MP cells. **g**) Real Time PCR array of differentiation markers comparing low MP to high MP cells. Note the enrichment of a number of markers for HPSCs and the downregulation of differentiation markers. *p < 0.05, ***p < 0.001

Given HSCs demonstrating distinct metabolic properties, we tested whether this metabolic footprint could enrich for HPSCs. Following flow cytometric separation of low and high MP cells, we utilized colony formation assay to test stem cell properties of low MP cells. Equal numbers of low and high MP cells were plated in methocult media for 12 days. Then, number and type of colonies were evaluated. Low MP cells had significantly (*p* < 0.001) higher colony formation compared to high MP cells (Fig. 3a). Additional classification of colony types showed that the low MP cells produced a higher percentage of CFU-GEMM type (mixed colonies) (Fig. 3b) with lower percentages of BFU-E (Fig. 3c) and CFU-GM (Fig. 3d) colonies than high MP cells. In addition to the *in vitro* assays, we also performed repopulation assays *in vivo* and demonstrated that low MP cells had much higher engraftment as compared to high MP cells. This provides the strong evidence that low MP cells are indeed enriched for HSPCs (Fig. 3e and Additional file 2: Fig. S2b). Furthermore, we evaluated the gene expression profile of low and high MP cells by PCR array. Similar to their mouse counterparts, low MP cells were characterized by enrichment of HPSC associated stem cell markers and diminished lineage differentiation markers when compared to high MP cells (Fig. 3f and g, respectively). These results indicate that low MP cells are enriched for human HPSCs.

### Expression of Hif-1α and Meis1 in human HPSCs

**Fig. 4:**
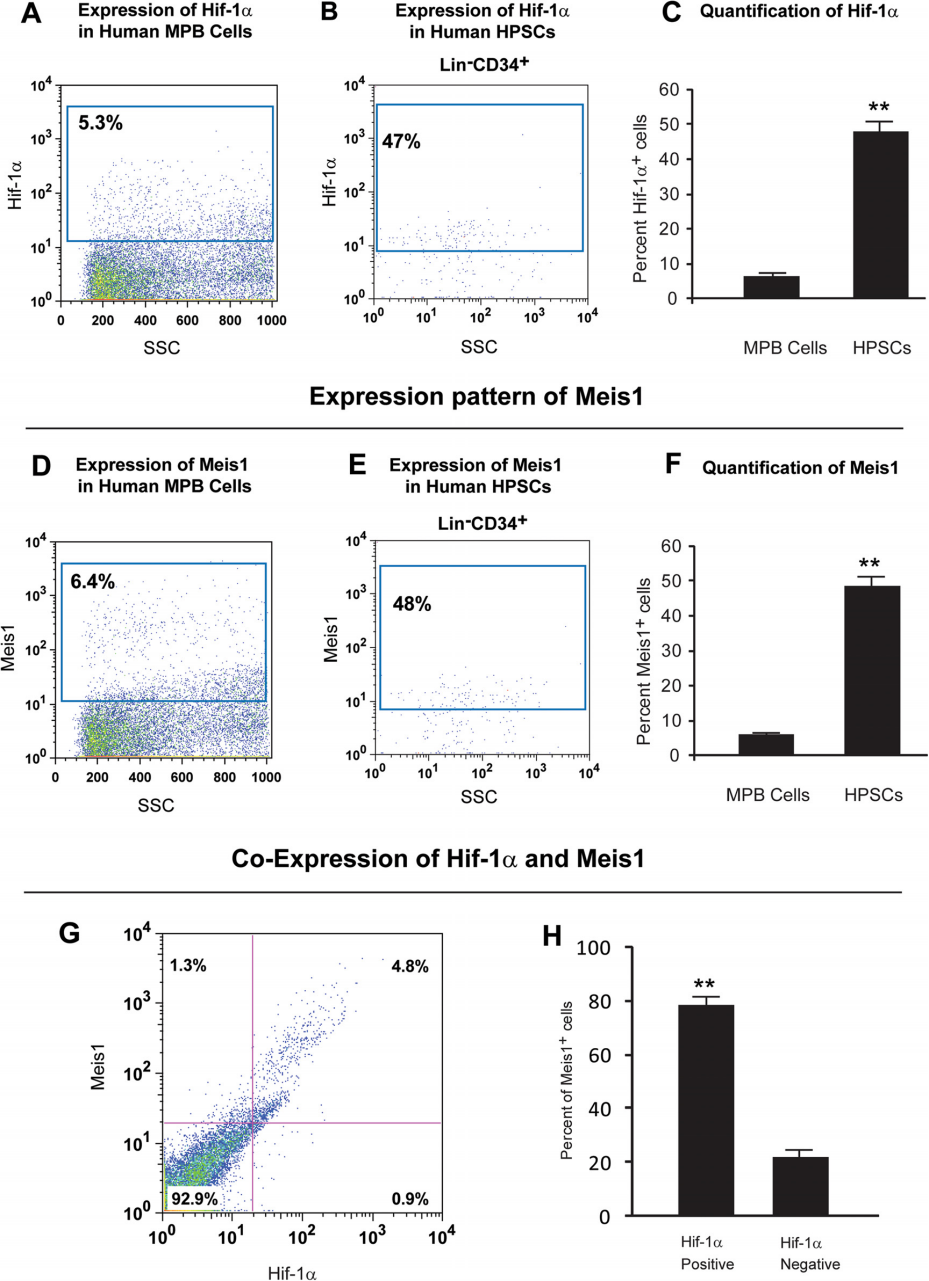
Expression profile of Hif-1α and Meis1 in human HPSCs. Expression pattern of Hif-1α **a**) The left panel shows expression of Hif-1α in human MPB cells. **b**) Expression of Hif-1α in human HPSCs. **c**) Quantification of Hif-1α expression. Note that while 5.3 % of the human MPB cells express Hif-1α, the significantly higher percentage (47 %) of HPSCs express Hif-1α (~9 fold) (*n* = 3). Expression pattern of Meis1 **d**) The left panel shows expression of Meis1 in human MPB cells. **e**) Expression of Meis1 in HPSCs. **f**) The right panel shows quantification of Meis1 expression. Note that while 6.4 % of the human mobilized peripheral blood cells express Meis1, 48 % of HPSCs express Meis1 (*n* = 3) (~7.5 fold). Co-expression of Hif-1α and Meis1 **g**) Flow cytometric colocalization of Hif-1α and Meis1 in the human MPB cells. Left panel shows a representative flow cytometry profile of Hif-1α and Meis1 expression. **h**) Quantification of the percent of Meis1^+^ cells. Note the majority of Meis1^+^ cells coexpress Hif-1α. **p < 0.01

In order to determine the molecular mechanism behind glycolytic phenotype of human HPSCs and the expression of Hif-1α in low MP MPB cells, we evaluated the expression pattern of Hif-1α and Meis1 in human MPB mononuclear cells and HPSCs. Meis1 is a transcription factor required for definitive hematopoiesis. We demonstrated that Meis1 transcriptionally regulates Hif-1α expression in mouse LT-HSCs [3]. However, whether Meis1 plays any role in the regulation of Hif-1α in human hematopoiesis was unknown. To address this question, we first determined the expression of Meis1 in human MPB mononuclear cells and HPSCs. Cells were fixed, permeabilized, and intracellularly stained by antibodies against Hif-1α and Meis1 followed by flow cytometry analysis. While about 5 % and 6 % of MPB mononuclear cells express Hif-1α and Meis1, respectively (Fig. 4a, Fig. 4d and Additional file 3: Fig. S3), a significantly higher percent of HPSCs express Hif-1α (47 %) (Fig. 4b-c and Additional file 4: Fig. S4) and Meis1 (48 %) (Fig. 4e-f and Additional file 4: Fig. S4). In addition, approximately 80 % of Meis1^+^ MPB cells are Hif-1α positive (Fig. 4g-h and Additional file 3: Fig. S3). This expression pattern was obtained after hours of MPB harvest, which suggests that expression of Hif-1α in the MPB cells is not secondary to the *in vivo* hypoxic niche in the bone marrow.

### Regulation of Hif-1α by Meis1

**Fig. 5:**
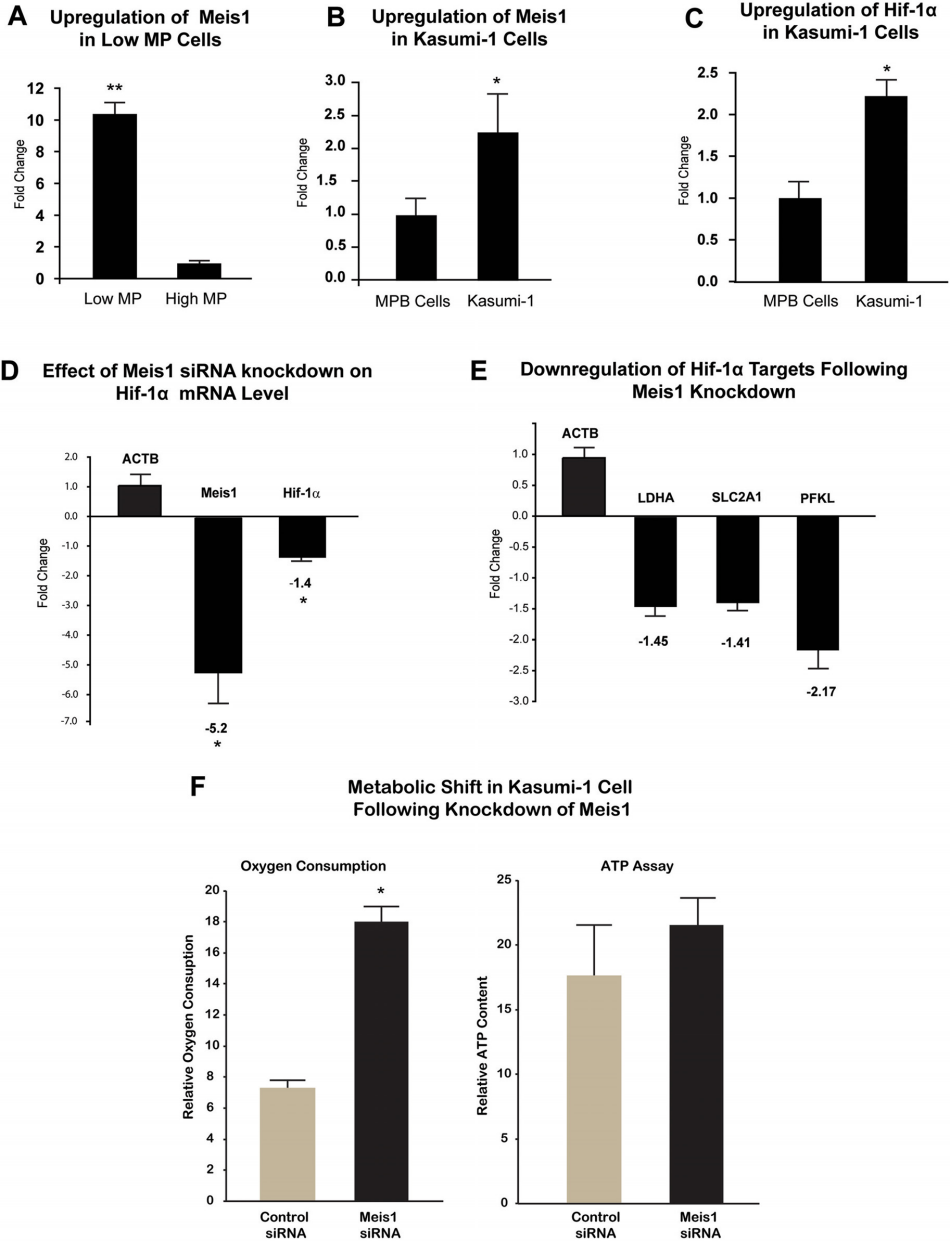
Transcriptional regulation of Hif-1α by Meis1. **a**) Real Time PCR of Meis1 in low and high MP cells demonstrates significantly higher levels of Meis1 expression in low MP cells. **b**) Upregulation of Meis1 in Kasumi-1 cells compared to human MPB Cells and **c**) Upregulation of Hif-1α in Kasumi-1 cells compared to human MPB Cells. **d**) Real time PCR of Meis1 and Hif-1α in Kasumi-1 cells following siRNA knockdown of Meis1. **e**) Real time PCR of lactate dehydrogenase (LDHA), Glut1 (SLC2A1) and phosphofructokinase (PFKL) following siRNA knockdown of Meis1. Note the significant downregulation of Hif-1α mRNA and Hif-1α target genes following siRNA knockdown of Meis1. **f**) Relative metabolic shift towards increased oxidative phosphorylation in Kasumi-1 cells following Meis1 knockdown: There is increased oxygen consumption and ATP levels following knockdown of Meis1 in Kasumi-1 cells. *n* = 3,*p < 0.05, **p < 0.01

Given the confirmed conserved Meis1 consensus binding sequence in the Hif-1α intron [1] and the Meis1 expression in human hematopoietic cells, we sought to determine whether Meis1 is required for Hif-1α expression in HPSCs. To this end, we used siRNA to knockdown Meis1 in Kasumi-1 cells (a human myeloid progenitor cell line known to express Hif-1α at normoxic conditions [23]). Upregulation of Meis1 or Hif-1α in low MP cells and in Kasumi-1 cells was confirmed by real time PCR (Fig. 5a to 5c respectively) prior to siRNA experiments. Scrambled Meis1 siRNA was used as control. 50 nM Meis1 siRNA resulted in significant decrease of Meis1 mRNA levels (5.2 +/− 1.0 fold reduction) (*p* < 0.05). Conistently, the knockdown efficiency of Meis1 was further confirmed by western blotting (Additional file 5: Fig. S5). This was associated with a significant downregulation of Hif-1α mRNA (Fig. 5d), as well as reduced expression of glycolytic genes downstream of Hif-1α including lactate dehydrogenase (LDHA), Glut1 (SLC2A1), and phosphofructokinase (PFKL) (Fig. 5e) (*p* < 0.05). Furthermore, knockdown of Meis1 in Kasumi-1 cells demonstrated a relative metabolic shift towards increased oxidative phosphorylation in Kasumi-1 cells as shown by increased oxygen consumption and ATP levels (Fig. 5f) compared to negative control. These results demonstrate that Meis1 is required for optimal expression of Hif-1α and its downstream target genes in human hematopoietic cells.

### Cooperation of Meis1 with Pbx1 and HoxA9 in the regulation of Hif-1α expression

**Fig. 6:**
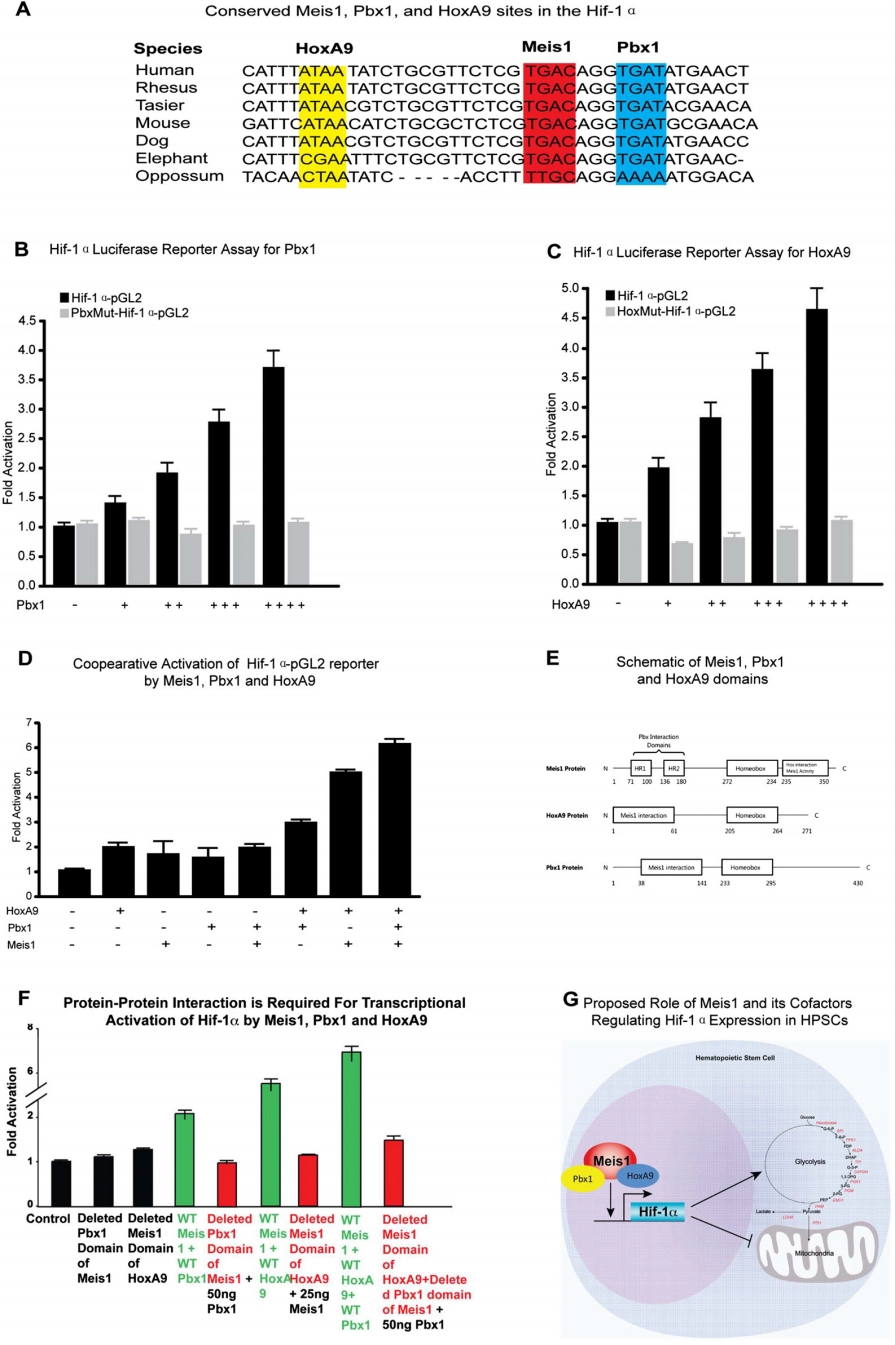
Cooperative role of Pbx1, HoxA9, and Meis1 for transcriptional regulation of Hif-1α. **a**) Figure shows conserved consensus Meis1, Pbx1, and HoxA9 motifs found on Hif-1α gene. Note the binding motifs found next to each other in close proximity and highly conserved. **b**) Luciferase reporter assays demonstrate dose-dependent transcriptional activation of Hif-1α (Hif-1α-pGL2 reporter) by Pbx1 and mutation of Pbx1 bindings sites abolished Hif-1α activation (PbxMut-Hif-1α-pGL2 reporter) **c**) Luciferase reporter assays demonstrate dose-dependent transcriptional activation of Hif-1α (Hif-1α-pGL2 reporter) by HoxA9 and mutation of HoxA9 bindings sites abolish Hif-1α activation (HoxMut-Hif-1α-pGL2 reporter) **d**) Luciferase reporter assays with different combinations of Meis1, Pbx1, and HoxA9 demonstrates (additive) cooperation of Meis1, Pbx1 and HoxA9 for activation of Hif-1α (Hif-1α-pGL2 reporter) **e**) Schematic of Meis1, Pbx1 and HoxA9 domains **f**) Meis1, Pbx1 and HoxA9 protein interaction were assessed by deletion of pbx1 interaction domain at Meis1 (71-100aa HR1 and 136-180aa HR regions) and Meis1 interaction domain of HoxA9 (1-61aa). Transcriptional activation by WT vectors, and vectors with deleted domains were evaluated using Hif-1α-pGL2 construct. Luciferase measurements were calculated as firefly luciferase units versus b-gal units. **g**) Schematic of proposed model showing regulation of Hif-1α expression by a complex of Meis1/Pbx1/HoxA9 proteins in hematopoietic stem cells

In addition to Meis1 binding motif in the Hif-1α intron, we identified conserved putative Pbx1 (“TGAT”) and HoxA9 (“ATAA”) binding motifs in close proximity to consensus Meis1 binding site (Fig. 6a). Meis1, Pbx1 and HoxA9 pose interaction domains or motifs that facilitate cooperation in gene activation. Given that Meis1 is known to cooperate with Pbx1 and HoxA9 in gene activation, we tested the role of Pbx1 and HoxA9 in the activation of Hif-1α. Using Hif-1α-pGL2 luciferase reporter, which includes conserved Meis1, Pbx1 and HoxA9 sites, we demonstrate a dose dependent activation of Hif-1α by Pbx1 (Fig. 6b) and HoxA9 (Fig. 6c) in addition to Meis1 as we showed earlier [3]. This activation demonstrates dependence on binding of Pbx1 or HoxA9 to its consensus binding sequences in the Hif-1α reporter as mutation of the seed sequences (PbxMut-Hif-1α-pGL2 or HoxMut-Hif-1α-pGL2 reporters, respectively) completely abolished the activation of Hif-1α by Pbx1 or HoxA9 respectively. To test co-operation of Meis1 with Pbx1 and HoxA9 in the Hif-1α expression, we performed further luciferase assays using different combinations of Meis1, Pbx1, and HoxA9 vectors. Here we demonstrate that Meis1, Pbx1 and HoxA9 cooperate in the activation of Hif-1α (Hif-1α-pGL2 reporter) (Fig. 6d).

To further characterize the interaction of Meis1 with Pbx1 and HoxA9 proteins in the activation of Hif-1α, we generated mutant Meis1 protein lacking Pbx1 interaction Motifs (PIM) and HoxA9 protein lacking Meis1 interaction domain (MID) (Fig. 6e). As shown Additional file 6: Fig. S6, we could stably detect the expression of Meis1, Meis1∆PIM, HoxA9 and Hoxa∆MID, which indicated that deletion mutants of the proteins were stable in our system. These mutant proteins allowed us to demonstrate that protein-protein interaction between Meis1 and Pbx1 as well as Meis1 and HoxA9 are required for transcriptional activation of Hif-1α luciferase reporter by Meis1, Pbx1 and HoxA9 (Fig. 6f). In summary, Hif-1α expression is regulated by cooperative activation of Meis1, Pbx1 and HoxA9 (Fig. 6g). This activation is dependent on consensus binding motifs located at Hif-1α intronic region.

## Discussion

Several studies indicated that human HPSCs reside in hypoxic niches and are capable of surviving upon hypoxic stress [1, 16, 17]. However, it is unclear if HPSCs are endowed with distinct metabolic properties that confer this hypoxic tolerance. In the current report, we show that human HPSCs are localized to a flow cytometry gate characterized by low mitochondrial potential (low MP). This population represents only 7–9 % of the G-CSF mobilized human peripheral blood cells, but is enriched for more than 70 % of human HPSCs as Lin^−^CD34^+^CD38^−^CD90^+^ cells. Moreover, we confirm this metabolic profile by demonstrating that human HPSCs have lower ATP content, lower rates of oxygen consumption, and use cytoplasmic glycolysis instead of mitochondrial oxidative phosphorylation. Interestingly, here we demonstrated that the glycolytic phenotype of HPSCs persists in circulating cells, suggesting that the metabolic phenotype of HSCs is a product of intrinsic regulatory mechanisms and not just their environment. Similar to their murine counterparts, this unique metabolic profile is associated with upregulation of Hif-1α. It is noteworthy that we found human HPSCs are localized to the low MP gate even after G-CSF mobilization to the peripheral blood, which indicates that HPSCs may have intrinsic mechanism for the regulation of their metabolic properties, other than their location in hypoxic niches.

Hif-1α as a master regulator of metabolism plays an essential role in various aspects of adaptation to hypoxic stress. Regulation of Hif-1α expression is well studied in terms of its posttranslational stabilization during hypoxia, however, little is known about its transcriptional regulation. Previous reports by our group and others indicate that Hif-1α is regulated at the transcription level not only at hypoxia but also normoxia [3, 22, 49–51]. Here we show that, the transcription factor Meis1 plays an important role in HPSC metabolism through transcriptional regulation of Hif-1α in cooperation with its cofactors Pbx1 and HoxA9. Meanwhile, we also noticed that anyone of Meis1, Pbx1 or HoxA9 was able to upregulate Hif-1α at transcription level (Fig. 6d). However, when one or more mutated factor(s) plus another WT factor did not transcriptionally upregulate Hif-1α (Fig. 6f). We speculated that the expression of mutant forms of the any protein might interrupt the action of any WT protein in transcriptional regulation of Hif-1α. This might indicate that normal expression levels of these factors play an important role in the synergistic effect in the expression of Hif-1α. Recently, our group recently demonstrated that Meis1 is an important regulator of postnatal cardiomyocyte cell cycle [52]. Intriguingly, we found that Pbx1 and HoxA9 were not expressed in postnatal cardiomyocytes, while other AbdB-like paralogue of Hox genes seem to have an expression pattern similar to Meis1. Therefore, it may be that the transcriptional activity of Meis1 in various compartments is regulated through differential expression pattern of its cofactors.

## Conclusions

In summary, our findings demonstrate the unique metabolic properties of human HPSCs as compared to human MPB mononuclear cells, as well as the transcriptional network that regulates their metabolic phenotype. The transcriptional regulation of Hif-1α by Meis1 and its cofactors, and the metabolic shift that follows loss of Meis1 suggest that the metabolic profile of human HPSCs is an intrinsic characteristic of these cells and not only a product of their hypoxic microenvironment. It is therefore important for future studies to determine the role of these unique metabolic properties in vitro human HPSC culture and expansion, as well as in designing therapeutic strategies for hematopoietic disorders.

## Methods

### Isolation of human mobilized peripheral blood (MPB) cells

Human G-CSF mobilized peripheral blood (MPB) was obtained from bone marrow transplant donors by plasmapheresis. Human cells were diluted and separated using ficoll to obtain the MPB mononuclear cell fraction. Then cells were suspended at 2–3 × 10^6^ cells/mL in DMEM media with 10 % fetal bovine serum for flow cytometry. The study was approved by the Institutional Review Board of affiliated hospital of UT Southwestern Medical Center (Dallas, USA) and Shanghai JiaoTong University School of Medicine (Shanghai, China). Written informed consent was obtained from each donor for his enrollment. All clinical investigation was conducted in accordance with the principles expressed in the Declaration of Helsinki.

### Flow cytometric profiling and separation of cells based on mitochondrial potential

Flow cytometric profiling and separation of human MPB cells based on their mitochondrial activity were performed by using mitotracker dyes as described previously [3]. Briefly, human MPB cells were enriched by lineage depletion using the BD IMag™ Human Lineage Cell Depletion Set (BD Biosciences) according to manufacturer’s instructions. The whole MPB fraction and Lin^−^CD34^+^CD38^−^CD90^+^ cells (30 min incubation on ice) were then profiled based on their mitochondrial activity after 15 min staining with mitotracker dyes (MitoTracker Red FM Cat# M22425 and MitoTracker Green Cat# M7514, Invitrogen) at 37 °C (2 × 10^6^ cells/mL, 200 nM mitotracker). Cell sorting based on high and low mitochondrial activity was performed using gates that separated the cells with those with the lowest 7–9 % of mitochondrial activity and equivalent number of cells with the high mitochondrial activity (named low and high mitochondrial potential (MP) cells).

### Colony forming assay and *in vivo* transplantation

Based on mitochondrial profile, colony forming cell (CFC) assays were performed on human G-CSF MPB cells. Same numbers of human high and low MP cells (3 × 10^4^ cells) were plated in each methocult plate (MethoCult® GF H4434, Stem Cell Technologies, USA). Viability was assessed with trypan blue as this is crucial that low MP gate often have dying cells with low mitochondrial activity. To minimize cell attachment, plates were precoated with 1 % agarose. After twelve days of culture, the total number of colonies (per plate) was counted and the types of colonies were quantified. Note CFU-GEMM colonies indicate mixed colonies whereas BFU-E and CFU-GM are measure of erythroid and myeloid progenitors, respectively. For *in vivo* transplantation, NOD-SCID mice were purchased from the Shanghai SLAC Laboratory Animal Co. Ltd. Five millions of FACS-sorted low MP or high MP cells in 50 μL of PBS were directly injected into the tibia as described previously [53]. Human engrafted cells in bone marrow were detected by using anti-human CD45 antibodies 2 months after transplantation.

### Oxygen consumption

Oxygen consumption was determined using the BD Oxygen Biosensor System, 384 well (Cat#353,834) according to manufacturer’s recommendations. MPB Cells and human HPSCs were separated as described above. Equal numbers of cells (5 × 10^4^ cells/well in 50 μL volume) were incubated up to 6 h in the provided 384 well plate prior to measurement at Fluostar Optima plate reader (BMG Labtech). Culture media lacking cells was used as a negative control and sodium sulfite (100 mM) was used as a positive control. Oxygen consumption is presented as relative units.

### ATP assay

HPSCs from human MPB Cells were isolated as described. Fifty thousand cells were used for each measurement. HPSCs and MPB Cells were centrifuged at 1200 g for 10 min. ATP standard curves were prepared using ATP concentrations between 10^−6^-10^−12^ M. Then, 50 μl of ATP standards and 50 μl cell lysates were quantified using ATP Bioluminescence Assay Kit HS II (Roche) using Fluostar Optima plate reader (BMG Labtech) following manufacturer instructions. Finally, data were normalized to cell count.

### Measurement of ^13^C lactate production

Cells were cultured in DMEM (Sigma D5030) supplemented with L-glutamine (4 mM), NaHCO_3_ (42.5 mM), HEPES (25 mM), dialyzed fetal calf serum (10 % v/v), Penicillin/Streptomycin and no glucose per well of 96-well plate. The medium was supplemented with double labeled 10 mM D-[1-^13^C, 6-^13^C]-glucose (Cambridge Isotope Labs) to allow all of the glucose-derived lactate pool to be labeled on C-3. Fifty thousand cells (each well) were cultured in 40 μL of medium for 12 h. After the culture, the cells were pelleted and an aliquot of 25 μL of the medium was transferred to a glass test tube with the internal standard (17.9 nmol of Sodium L-[^13^C_3_]-Lactate, Cambridge Isotope Labs). Lactate was extracted by sequential addition of 1 mL of methanol, chloroform and water followed by vortexing and centrifugation at 2000 rpm for 5 min. The aqueous phase was evaporated, derivatized with 100 μL Tri-Sil reagent (Pierce) for 30 min at 42 °C, and analyzed by gas chromatography–mass spectrometry. A three-point standard curve was also prepared using mixtures of un-enriched lactate and L-[3-^13^C_3_]-lactate (Cambridge Isotope Labs). Lactate abundance was determined by monitoring m/z at 117 (un-enriched), 118 (lactate containing ^13^C from glucose) and 119 (internal standard). The areas of 117 and 118 were summed and corrected against the 119 area to calculate total lactate abundance. To determine the atom percent excess (APE), the 117 and 118 areas were first corrected against the 119 abundance to account for inter-sample variability of extraction. Then the corrected ratio of 118/(117 + 118) was determined and compared to the standard curve. Finally, the APE was multiplied by the total nmoles lactate to determine the nmoles of ^13^C-lactate produced. The final results were corrected for total cellular ATP concentration.

### Metabolic assays with seahore XF96

Oxygen consumption and lactate generation were measured using the Seahorse XF96 extracellular flux analyzer as previously described [54]. Briefly, three replicate wells of 3 × 10^5^ low MP or high MP cells per well were seeded overnight in 96-well XF96 well plates coated with BD Cell-Tak (BD Biosciences) in serum-free culture medium containing 20 ng/ml SCF and 20 ng/ml TPO. One hour prior to analysis, the medium was replaced by unbuffered DMEM and the well plates were incubated to 37°C for pH stabilization. Analyses were performed both at basal conditions and after injection of oligomycin (0.5 μM for OCR or 2 μM for ECAR), FCCP (2 μM), Antimycin A (0.5 μM), Glucose (10 mM), and 2-DG (100 mM).

### Meis1 knockdown

Meis1 siRNA knockdown was carried out in Kasumi-1 cells (human myeloid progenitor cell line known to express Hif-1α at normoxic conditions [23]). Upregulation of Meis1 in low MP cells and Kasumi-1 cells compared to human MPB cells were confirmed by real time PCR. Kasumi-1 cells were diluted to a density of 200,000 cells per mL in RPMI media supplemented with 20 % FBS and antibiotics. The cells were centrifuged for 10 min at 1200 g at 4 °C and suspended in OPTIMEM (Invitrogen). The cells were then plated into 6-well plates. 12 μL of Hiperfect (Qiagen) and siRNA (50nM of siRNA per 750,000 cells/well) were incubated in 200 μL of OPTIMEM (Invitrogen) for 20 min at RT. Silencer Select Pre-designed siRNAs (Applied Biosystems, Ambion) for Meis1 were diluted into 50nM stocks (siRNA ID# s8662: 5’-GGCAUCUACUCGUUCAGGAtt-3’ and 5’-UCCUGAACGAGUAGAUGCCgt-3’). siRNA were added to cells dropwise and incubated for 6 h. After 6 h incubation, 2.5 mL RPMI medium was added (20 % FBS and antibiotics) and plates were incubated under normal growth conditions for 48 h.

### Expression profile of low and high MP cells

PCR arrays (HSC and hypoxia primer sets, SABiosciences) were performed to study HPSC and hypoxia inducible gene expression profile of low and high MP cells. RNA was isolated from low and high MP cells by TRIzol method (Invitrogen, Carlsbad, CA) following manufacturer instructions. cDNA was generated by using an RT-PCR kit (SA biosciences, Maryland, USA). Real-Time PCR was performed using Human Real-Time Syber Green PCR Mix (SuperArray) on an ABI Prism 7700 Sequence Detector (Applied Biosystems). The data was analyzed using the ΔΔCt method. Fold change was calculated as difference in gene expression between low and high MP cells.

### Real time PCR for Meis1, Hif-1α and Hif-1α related genes

Total RNA was isolated using TRIzol reagent (Invitrogen). cDNA was generated by following the recommended protocol for SuperScript II Reverse Transcriptase (Invitrogen) using 2 μg total RNA. Real time PCR was performed with SyberGreen (Applied Biosystems) on ABI Prism 7700 Sequence Detector (Applied Biosystems) using primers at Table 1.

**Table 1:** Primers used in this study

Gene [GenBank: Accession Numbers]	Forward Primer	Reverse Primer
Meis1 [NM_002398.2]	5’-ACGCTTTTTGTGACGCTTTT-3’	5’-TCACACAGTGGGGACAACAG-3’
Hif-1α [NM_001243084.1]	5’-GAAGTGGCAACTGATGAGCA-3’	5’-GCGCGAACGACAAGAAA-3’
LDHA [NM_001135239.1]	5’-GGAGATCCATCATCTCTCCC-3’	5’-GGCCTGTGCCATCAGTATCT-3’
SLC2A1 [NM_006516.2]	5’-GGCATTGATGACTCCAGTGTT-3’	5’-ATGGAGCCCAGCAGCAA-3’
PFKL [NM_001002021.2]	5’-GATGATGTTGGAGACGCTCA-3’	5’-GGTGCCAAAGTCTTCCTCAT-3’
SIAH2 [NM_005067.5]	5’-GTTTCTCCGTATGGTGCAGG-3’	5’-TCAGGAACCTGGCTATGGAG-3’
PHD2 [NM_022051.2]	5’-GTTCCATTGCCCGGATAAC-3’	5’-CGACCTGATACGCCACTGTA-3’
VDU2 [NM_001008563.4]	5’-TAGGGGTCTGGAGTGAGTGG-3’	5’-CTCTGAGCATTGGCGACC-3’

### Intracellular detection of Hif-1α and Meis1 in HPSCs

Human MPB cells and human HPSCs underwent fixation with 4 % paraformaldehyde for 10 min at room temperature. After permeabilization (0.01 % Triton) and serum block, cells were incubated overnight with primary antibodies; 1:50 dilution of anti-Hif-1α (Cat#610958, BD Transcduction Laboratories) and 1:50 dilution of anti-Meis1 (sc-10599, Santa Cruz Biotechnology). Staining was assessed by flow cytometry after incubation with corresponding fluorophore-conjugated secondary antibody.

### Generation of Meis1 lacking Pbx1 interaction motifs and HoxA9 lacking Meis1 interaction domain constructs

Meis1 and Pbx1 protein interaction were assessed by deletion of Pbx1 interaction domain of Meis1. For that purpose, we generated Meis1 lacking aminoacids for Pbx1 interaction domain (both 71–100 aa HR1 and 136-180aa HR regions) by polymerase chain reaction (PCR) from pCMV-SPORT6-Meis1 (MMM1013-7512739, Openbiosystems) using primers (NotI site were inserted for screening) Meis1dPIM(71–130)F: 5’- ATCTATcatgcggccgcAAAATGCCTATCGATTTGGTGATAG-3’ and Meis1dPIM(71–130)R: 5’-ATCTATcatgcggccgcTAAAGCGTCATTGACCGAGGAACCC-3’.

Meis1 and HoxA9 protein interaction were studied by co-tranfection of Meis1 with HoxA9 lacking Meis1 interaction domain (MID). HoxA9 lacking aminoacids for Meis1 interaction domain (1–61) were generated by PCR from pCMV-SPORT6-HoxA9 (MMM1013-9200573, Openbiosystems) using primers (BamHI site were inserted for screening) HoxA9dN(1–61)F: 5’-ATCTATcatggatccCATTGCAGTAGCCCGCGCCTG GCCGG-3’ and HoxA9dN(1–61)R: 5’-ATCTATcatggatccGCGGTGTTTGGTGCCTCGTGGAACCC-3’.

### Luciferase assays

Transcriptional activation of Hif-1α reporter by Meis1 (pCMV-SPORT6-Meis1, Cat#MMM1013-7512739,Openbiosystems), Meis1 lacking Pbx1 interaction domain (Meis1∆PIM), HoxA9 (pCMV-SPORT6-HoxA9, Cat# MMM1013-9200573, Openbiosystems), HoxA9 lacking Meis1 interaction domain (Hoxa∆MID) and Pbx1 (pCMV-SPORT6-Pbx1, Cat# MMM1013-63227,Openbiosystems), were evaluated using Hif-1α-pGL2 or PbxMut-Hif-1α-pGL2 (having Pbx1 binding site mutated) or HoxMut-Hif-1α-pGL2 (having HoxA9 binding site mutated) reporter constructs as indicated in the figures. 0.8 μg of Hif-1α-pGL2 or PbxMut-Hif-1α-pGL2 or HoxMut-Hif-1α-pGL2 reporter construct were co-transfectedwith different doses of the Meis1, Meis1∆PIM, HoxA9, Hoxa∆MID and Pbx1 expression vectors and 0.2 μg of pCMV-LacZ (internal control) into COS cells using lipofectamine transfection reagent (Invitrogen). At 48 h after transfection, cell lysate were prepared and quantified for firefly luciferase activity using a luciferase reporter system (Promega). Luciferase measurements were calculated as firefly luciferase units versus b-gal units. Transcriptional activation was compared to basal luciferase levels in cells transfected with Hif-1α-pGL2 or PbxMut-Hif-1α-pGL2 or HoxMut-Hif-1α-pGL2 and empty pGL2. To test Pbx1 site specificity, Pbx1 binding site TGAT at Hif-1α-pGL2 reporter were mutated using site directed mutagenesis kit (Strategene) from Hif-1α-pGL2 vector with the following primers: PbxMut-Hif-1α-F:5’-CAATTTCTACAAACTTGTGTTTGCCG ACCCTGTCAGGAGAGCCCAGACGTTA-3’ and PbxMut-Hif-1α-R: 5’-TAACGTCTGGGCTCTCCTGACAGGGTC GGCAAACACAAGTTTGTAGAAATTG-3’. Similarly, to test HoxA9 site specificity, HoxA9 binding site ATAA at Hif-1α reporter using site directed mutagenesis kit from Hif-1α-pGL2 vector with the following primers: HoxMu-Hif-1α-F: 5’-CCTGTCAGGAGAGCCCAGACGCCGCGTATCAATATGTGGCTG CCTC-3’ and HoxMut-Hif-1α-R: 5’-GAGGCAGCCACATATTGATACGCGGCGTCTG GGCTCTCCTGACAGG-3’.

### Western blotting and apoptosis assay

Plasmids of pCMV-SPORT6-Meis1, Meis1∆PIM, pCMV-SPORT6-HoxA9 and Hoxa∆MID were transfected into 293 T cells. Whole cell lysates were electrophoresed on 8–10 % sodium dodecyl sulfate polyacrylamide gels and transferred onto polyvinylidene difluoride membranes (Millipore). The membranes were incubated with primary antibodies overnight at 4 °C followed by incubation with appropriate horseradish peroxidase-conjugated secondary antibodies. The following antibodies were used: anti-Meis1 (Abcam), anti-HoxA9 (Proteintech) and anti-β-actin (Calbiochem). For analysis of apoptosis, cells were stained with PE conjugated anti-annexin V and 7-AAD (BD Pharmingen) according to the manufacturer’s instructions.

### Statistical analysis

Results are expressed as mean ± SEM and a 2-tailed Student *t* test was used to determine the level of significance. *p* < 0.05 was considered statistically different.

## Additional files

Additional file 1: Figure S1. Clustering and quantification of HSC frequency and lineages in Low or High MP cells. A) Quantification of HSC frequency in low MP and high MP cells: HSC (Lin^−^CD34^+^CD38^−^CD90^+^) frequency in low MP cells was 1.23 % (4.94 %*0.249 = 1.23 %), which was around 15-fold higher compared to high MP cells (0.332 %*0.252 = 0.084 %). B) Gating strategy showing clustering of human HPSCs into low and high MP gates. Human HPSCs clustered into low MP and high MP cells, where HSPCs were mostly located in low MP gate. C-D) Lineage distribution was measured in low and high MP cells by flow cytometric analysis with anti-CD19 and anti-Mac-1 antibodies. (PDF 711 kb) Additional file 2: Figure S2. A) Quantification of the percentage of Annexin V^+^ cells in low MP and high MP cells at normoxia or hypoxia. B) Representative flow cytometric analysis of repopulation of low MP and high MP cells. (PDF 324 kb) Additional file 3: Figure S3. Gating stragegy for Meis1 and Hif-1α intracellular staining in human MPB Cells (Related to Fig. 4A, 4D, and 4G). (PDF 730 kb) Additional file 4: Figure S4. Gating stragegy for Meis1 and Hif-1α intracellular staining in human HPSCs (Lin^−^CD34^+^) (Related to Fig. 4B and 4E). (PDF 442 kb) Additional file 5: Figure S5. Knockdown efficiency of Meis1 by siRNA was examined by western blotting. (PDF 160 kb) Additional file 6: Figure S6. WT and Deletion mutants (Meis1, Meis1∆PIM, HoxA9 and Hoxa∆MID) were overexpressed in 293T cells and detected by western blotting with anti-Meis1 and anti-HoxA9 antibodies. Both WT and deletion mutants of Meis1, HoxA9 were stably detected. (PDF 461 kb)

## Abbreviations

HSCs: Hematopoietic stem cells
HPSCs: Human hematopoietic progenitor and stem cells
Hif-1α: Hypoxia-inducible factor 1-alpha
Meis1: Meis homeobox 1
MP: Mitochondrial potential
Pbx1: Pre-B-cell leukemia transcription factor 1
HoxA9: Homeobox A9
