# Supplementary material for: Hypoxic metabolism in human hematopoietic stem cells
Source: Cell Biosci. 2015 Jul 17;5:39. doi: 10.1186/s13578-015-0020-3 (PMC4517642; doi:10.1186/s13578-015-0020-3)

sFigure 1

A Quantification of HSC frequency in Low MP and High MP cells

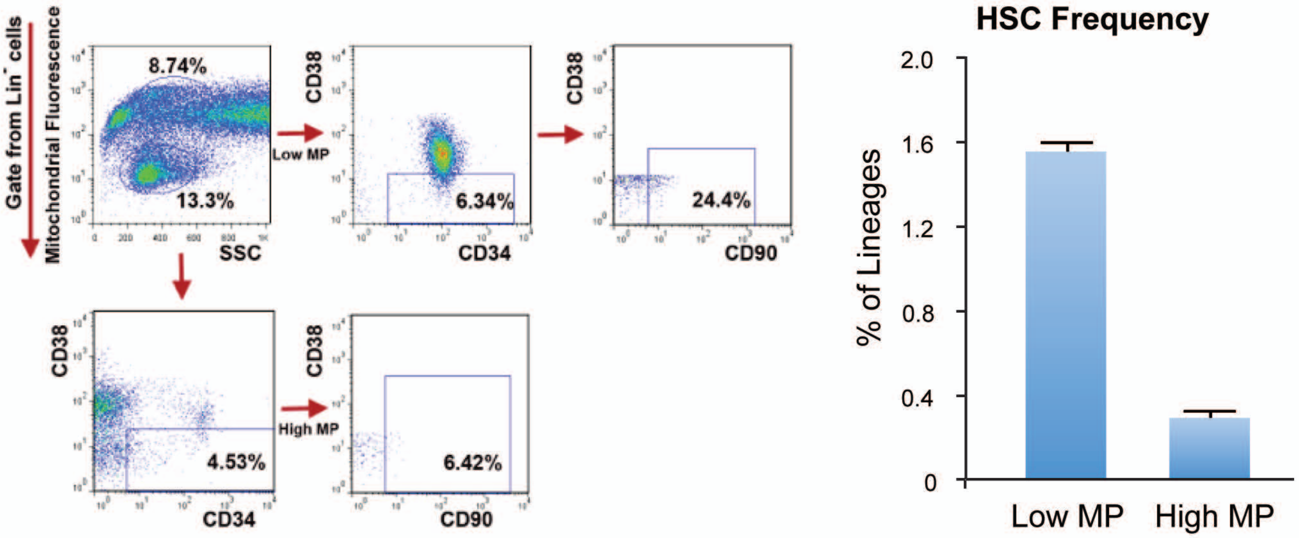

B Human HPSCs clusters into Low MP and High MP cells

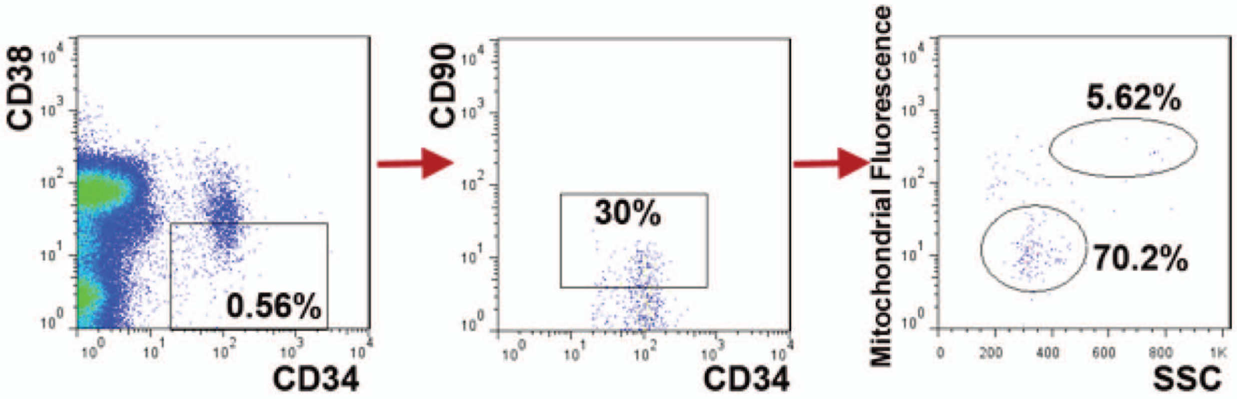

C CD19 expression

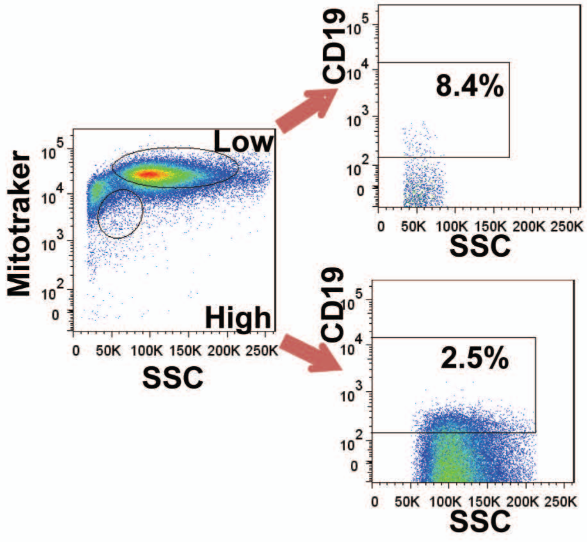

D Mac-1 expression

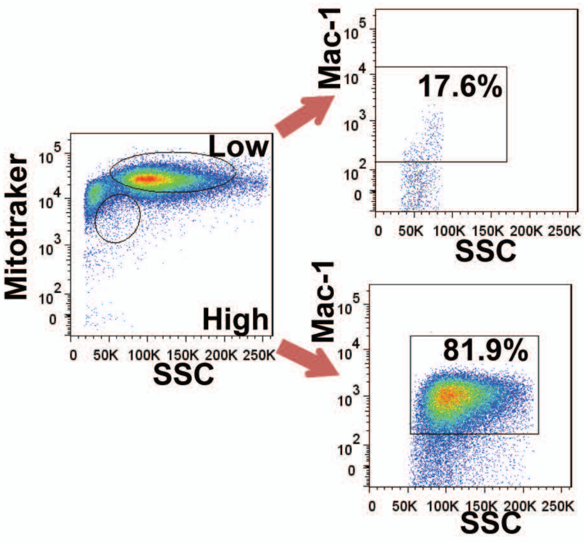

Supplement: Additional file 1: Figure S1. — Clustering and quantification of HSC frequency and lineages in Low or High MP cells. A) Quantification of HSC frequency in low MP and high MP cells: HSC (Lin−CD34+CD38−CD90+) frequency in low MP cells was 1.23 % (4.94 %*0.249 = 1.23 %), which was around 15-fold higher compared to high MP cells (0.332 %*0.252 = 0.084 %). B) Gating strategy showing clustering of human HPSCs into low and high MP gates. Human HPSCs clustered into low MP and high MP cells, where HSPCs were mostly located in low MP gate. C-D) Lineage distribution was measured in low and high MP cells by flow cytometric analysis with anti-CD19 and anti-Mac-1 antibodies. (PDF 711 kb) [file 13578_2015_20_MOESM1_ESM.pdf]
