# Supplementary figures and images for: Hypoxic metabolism in human hematopoietic stem cells
Source: Cell Biosci. 2015 Jul 17;5:39. doi: 10.1186/s13578-015-0020-3 (PMC4517642; doi:10.1186/s13578-015-0020-3)

sFigure 2

A Survival of Low MP Cells at Hypoxia

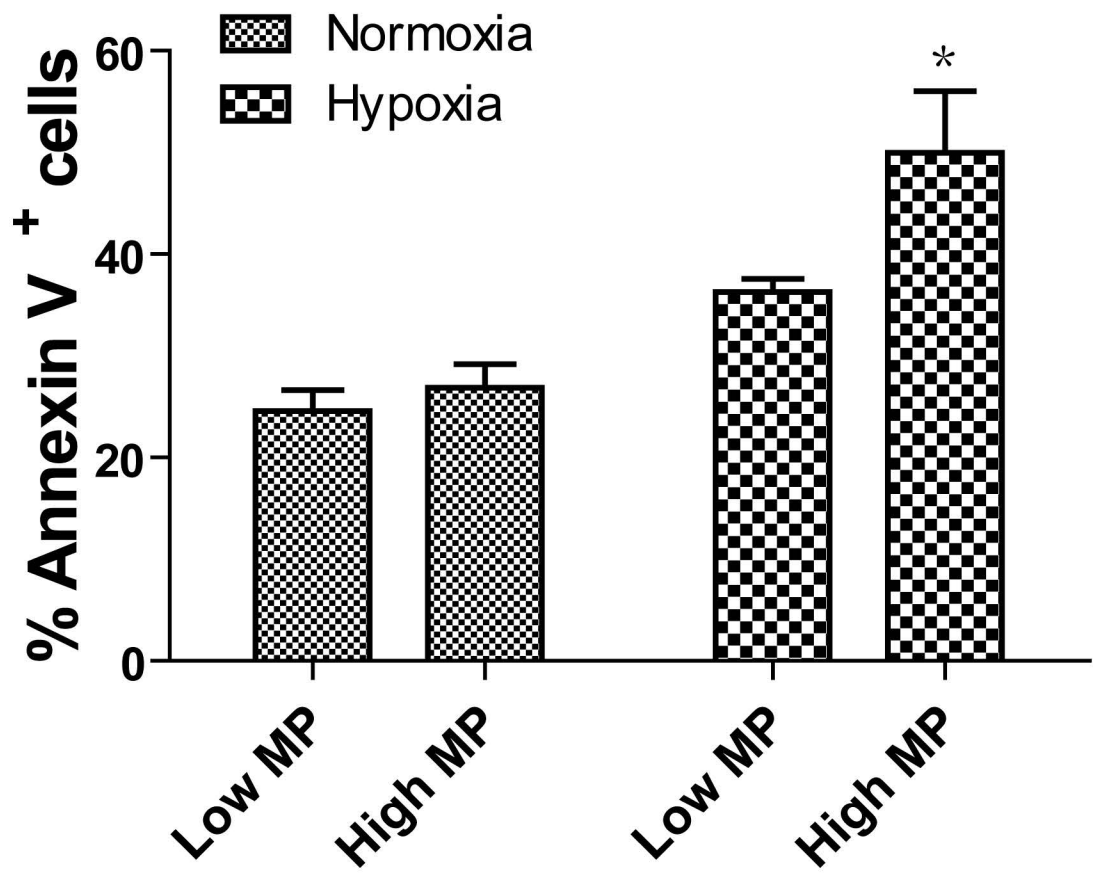

B Repopulation of Low/High MP Cells

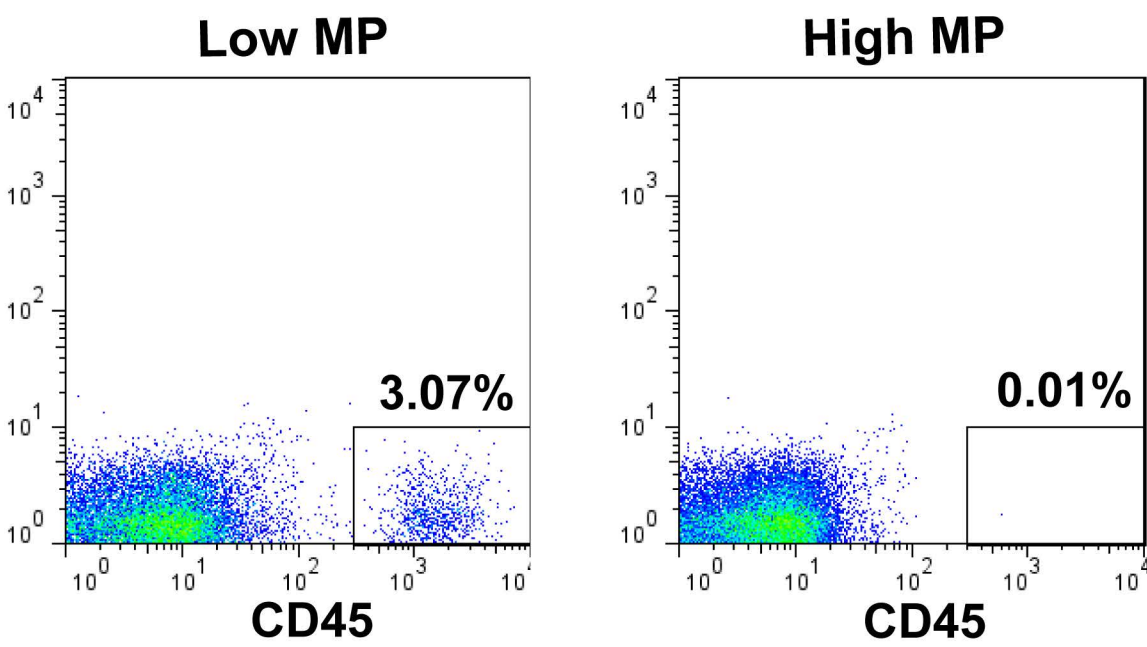

Supplement: Additional file 2: Figure S2. — A) Quantification of the percentage of Annexin V+ cells in low MP and high MP cells at normoxia or hypoxia. B) Representative flow cytometric analysis of repopulation of low MP and high MP cells. (PDF 324 kb) [file 13578_2015_20_MOESM2_ESM.pdf]

**sFigure 5 Knockdown efficiency of Meis1 by siRNA**

**Control siRNA**

**Meis1 siRNA**

**Meis1**

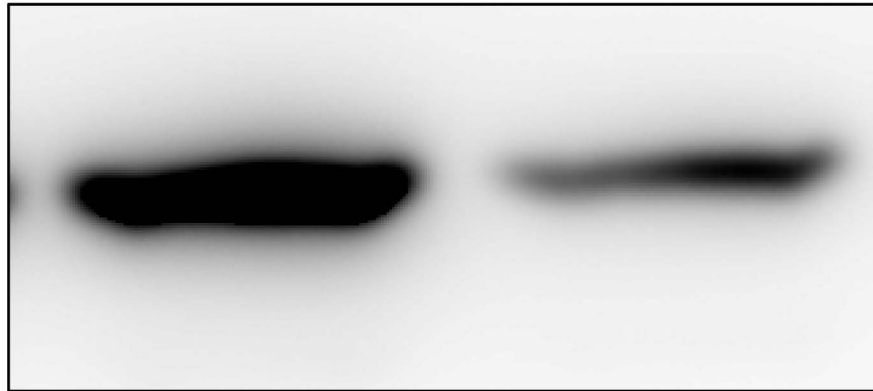

**GAPDH**

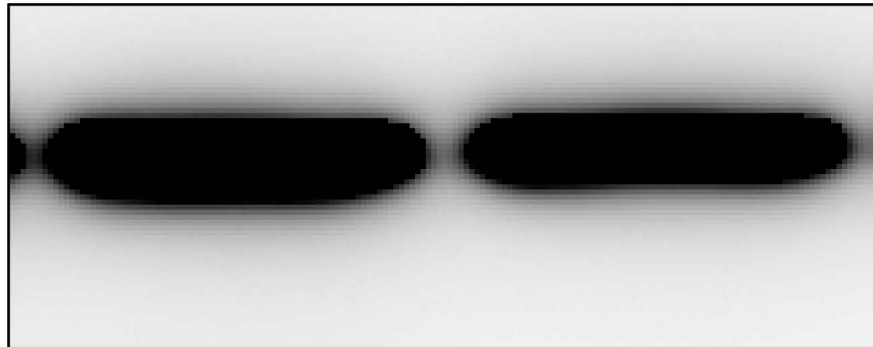

Supplement: Additional file 5: Figure S5. — Knockdown efficiency of Meis1 by siRNA was examined by western blotting. (PDF 160 kb) [file 13578_2015_20_MOESM5_ESM.pdf]
