# Supplementary material for: Hypoxic metabolism in human hematopoietic stem cells
Source: Cell Biosci. 2015 Jul 17;5:39. doi: 10.1186/s13578-015-0020-3 (PMC4517642; doi:10.1186/s13578-015-0020-3)

sFigure 3 Gating stragegy for Meis1 and Hif-1α intracellular staining in human MPB Cells (Related to Figure 4A, 4D, and 4G)

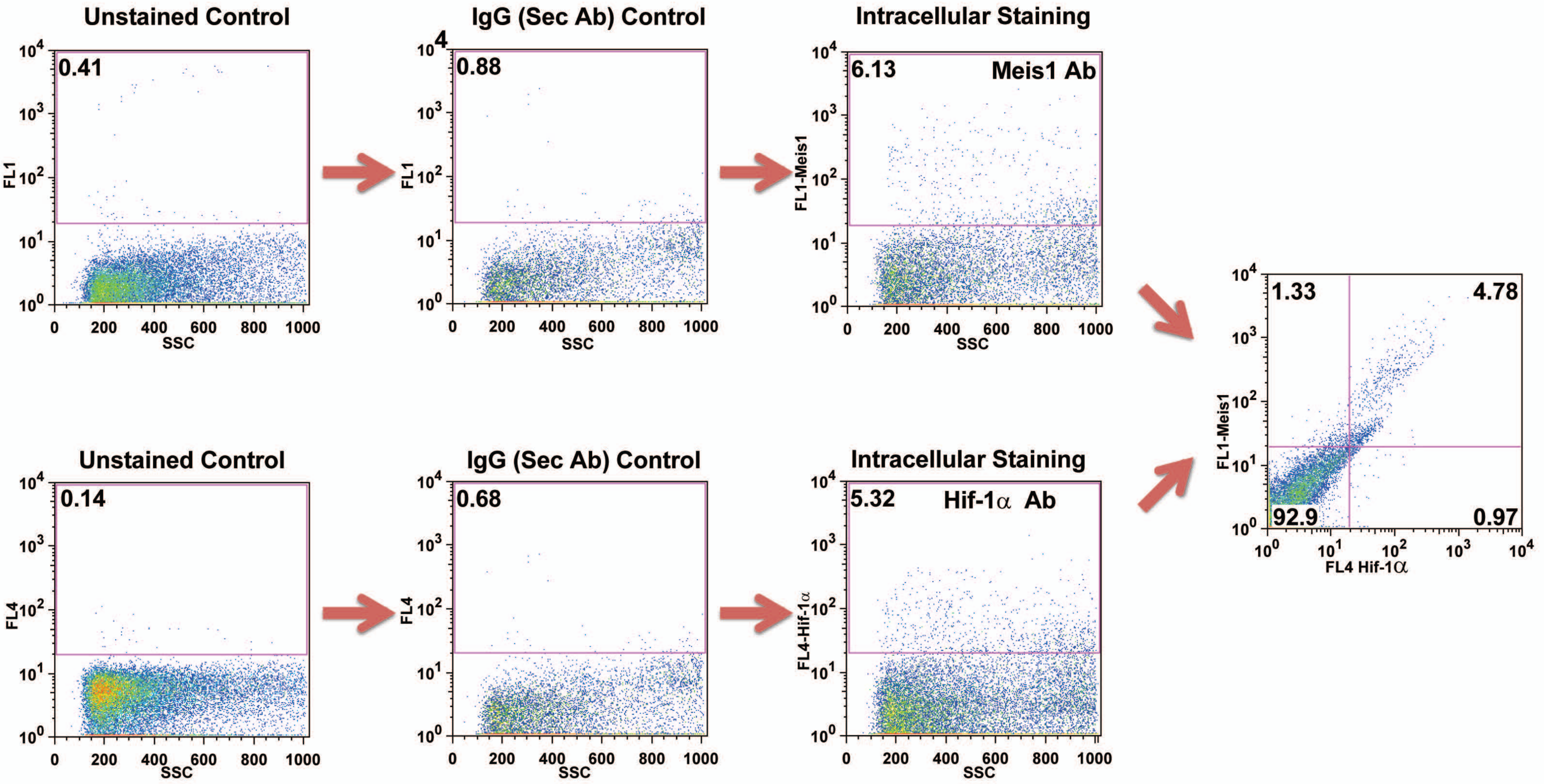

Supplement: Additional file 3: Figure S3. — Gating stragegy for Meis1 and Hif-1α intracellular staining in human MPB Cells (Related to Fig. 4A, 4D, and 4G). (PDF 730 kb) [file 13578_2015_20_MOESM3_ESM.pdf]
