# Supplementary material for: Hypoxic metabolism in human hematopoietic stem cells
Source: Cell Biosci. 2015 Jul 17;5:39. doi: 10.1186/s13578-015-0020-3 (PMC4517642; doi:10.1186/s13578-015-0020-3)

sFigure 4 Meis1 and Hif-1α intracellular staining in human HPSCs (Lin$^-$ CD34$^+$) (Related to Figure 4B and 4E)

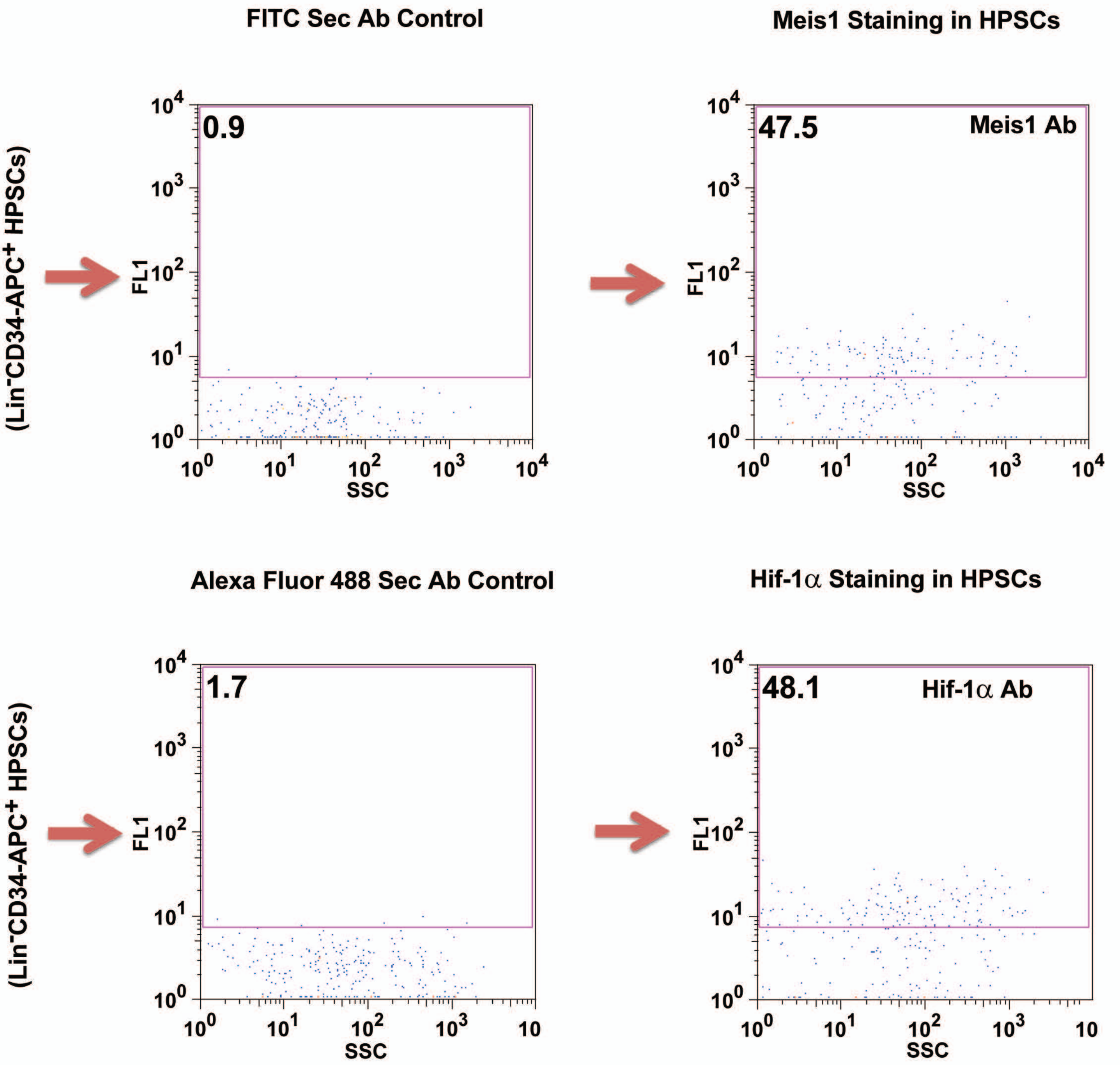

Supplement: Additional file 4: Figure S4. — Gating stragegy for Meis1 and Hif-1α intracellular staining in human HPSCs (Lin−CD34+) (Related to Fig. 4B and 4E). (PDF 442 kb) [file 13578_2015_20_MOESM4_ESM.pdf]
