# Supplementary material for: Hypoxic metabolism in human hematopoietic stem cells
Source: Cell Biosci. 2015 Jul 17;5:39. doi: 10.1186/s13578-015-0020-3 (PMC4517642; doi:10.1186/s13578-015-0020-3)

sFigure 6 Expression of Meis1 and HoxA9 deletion mutants

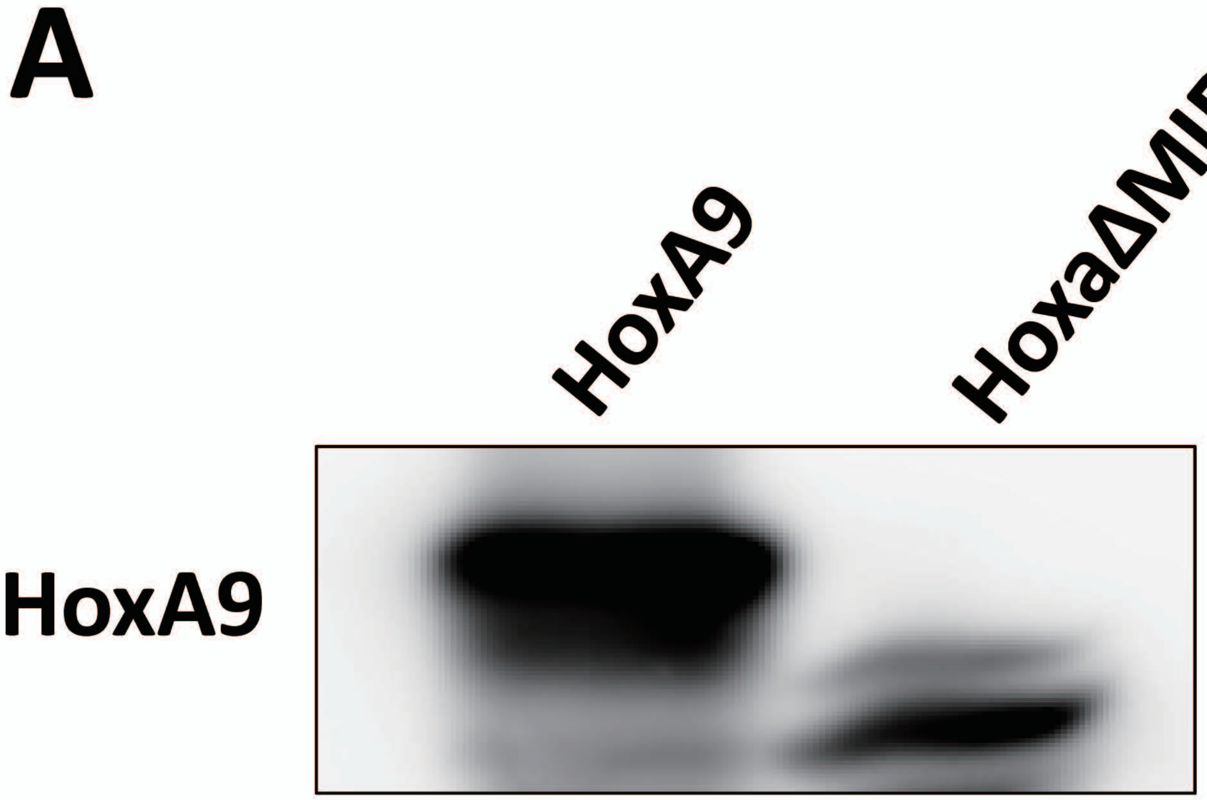

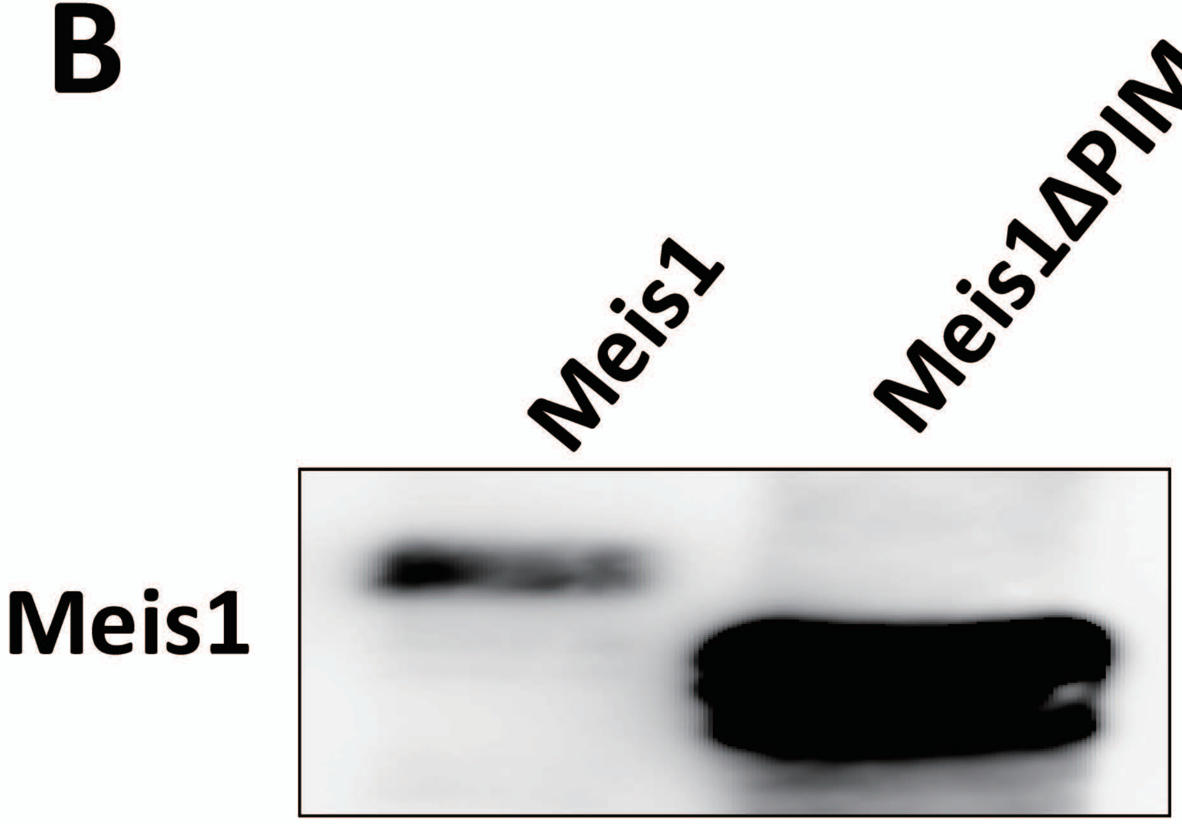

Supplement: Additional file 6: Figure S6. — WT and Deletion mutants (Meis1, Meis1∆PIM, HoxA9 and Hoxa∆MID) were overexpressed in 293T cells and detected by western blotting with anti-Meis1 and anti-HoxA9 antibodies. Both WT and deletion mutants of Meis1, HoxA9 were stably detected. (PDF 461 kb) [file 13578_2015_20_MOESM6_ESM.pdf]
